# A retrospective analysis of cardiovascular adverse events associated with immune checkpoint inhibitors

**DOI:** 10.1186/s40959-021-00106-x

**Published:** 2021-05-28

**Authors:** Jessica Castrillon Lal, Sherry-Ann Brown, Patrick Collier, Feixiong Cheng

**Affiliations:** 1 Genomic Medicine Institute, Lerner Research Institute, Cleveland Clinic, Cleveland, OH 44195 USA; 2 Department of Molecular Medicine, Cleveland Clinic Lerner College of Medicine, Case Western Reserve University, Cleveland, OH 44195 USA; 3 Cardio-Oncology Program, Division of Cardiovascular Medicine, Medical College of Wisconsin, Milwaukee, WI 53226 USA; 4 Robert and Suzanne Tomsich Department of Cardiovascular Medicine, Cleveland Clinic, Sydell and Arnold Miller Family Heart and Vascular Institute, Cleveland, OH 44195 USA; 5 Case Comprehensive Cancer Center, Case Western Reserve University School of Medicine, Cleveland, OH 44106 USA

**Keywords:** Cardio-oncology, Cardiotoxicity, Immune checkpoint inhibitors, Immunotherapy, Myocardial infarction, Myocarditis

## Abstract

**Background:**

Modern therapies in oncology have increased cancer survivorship, as well as the incidence of cardiovascular adverse events. While immune checkpoint inhibitors have shown significant clinical impact in several cancer types, the incidence of immune-related cardiovascular (CV) adverse events poses an additional health concern and has been reported.

**Methods:**

We performed a retrospective analysis of the FDA Adverse Event Reporting System data of suspect product reports for immunotherapy and classical chemotherapy from January 2010–March 2020. We identified 90,740 total adverse event reports related to immune checkpoint inhibitors and classical chemotherapy.

**Results:**

We found that myocarditis was significantly associated with patients receiving anti-program cell death protein 1 (PD-1) or anti-program death ligand 1 (PD-L1), odds ratio (OR) = 23.86 (95% confidence interval [CI] 11.76–48.42, (adjusted *p*-value) *q* <  0.001), and combination immunotherapy, OR = 7.29 (95% CI 1.03–51.89, *q* = 0.047). Heart failure was significantly associated in chemotherapy compared to PD-(L)1, OR = 0.50 (95% CI 0.37–0.69, *q* <  0.001), CTLA4, OR = 0.08 (95% CI 0.03–0.20, *q* <  0.001), and combination immunotherapy, OR = 0.25 (95% CI 0.13–0.48, *q* <  0.001). Additionally, we observe a sex-specificity towards males in cardiac adverse reports for arrhythmias, OR = 0.81 (95% CI 0.75–0.87, *q* <  0.001), coronary artery disease, 0.63 (95% CI 0.53–0.76, *q* <  0.001), myocardial infarction, OR = 0.60 (95% CI 0.53–0.67, *q* <  0.001), myocarditis, OR = 0.59 (95% CI 0.47–0.75, *q* <  0.001) and pericarditis, OR = 0.5 (95% CI 0.35–0.73, *q* <  0.001).

**Conclusion:**

Our study provides the current risk estimates of cardiac adverse events in patients treated with immunotherapy compared to conventional chemotherapy. Understanding the clinical risk factors that predispose immunotherapy-treated cancer patients to often fatal CV adverse events will be crucial in Cardio-Oncology management.

**Supplementary Information:**

The online version contains supplementary material available at 10.1186/s40959-021-00106-x.

## Introduction

Immune checkpoint inhibitors (ICIs) have increased cancer survivorship and are now standard of care for numerous cancer types [1–3]. Antibodies targeting programmed cell death protein (PD-1), programmed death-ligand (PD-L1), and cytotoxic T-lymphocyte-associated protein 4 (CTLA4) work to re-invigorate the immune system to recognize and lyse tumors. ICIs have now gained 67 FDA approvals in over 17 cancer types, and two tissue agnostic conditions [4, 5]. However, adverse events (AE) are common in patients receiving anti-CTLA4 (89%) and anti-PD-1 or PD-L1 (PD-(L)1) (74%) therapies. Virtually all patients receiving more than one ICI experience adverse events (90%) [6]. While cardiac adverse events comprise less than 1% of all AE, they are disproportionally more lethal [6]. It is unclear whether this low incidence results from a lack of reporting or misdiagnosis in part due to heterogeneity in clinical presentation. Cardiac AE encompasses a diverse set of disorders in the heart including myocarditis, pericarditis, arterial vascular disease, venous thromboembolism, pulmonary hypertension, arrhythmias, and heart failure. Further complicating the differential diagnosis and reporting, is the fact that more than one immune-related adverse event may occur with another [7]. Which clinical features predispose cancer patients to immune-associated cardiac AE remains poorly understood. As increased numbers of new targeted and immune-based therapies enter the market, the management of cancer patients continues to become more complicated with an increased need for predictive biomarkers [8, 9].

Pharmacovigilance studies prior to 2018 have raised awareness of the incidence of cardiac AE following ICI, leading to more case reports in the past 2 years (Additional File 1: Supplemental Table 1) [8, 10, 11]. Therefore, we sought to analyze and characterize cardiac adverse events associated with ICI in recent years. To achieve this, we mined case reports from the FDA Adverse Event Reporting System to determine if ICI-associated cardiac adverse events are disproportionally more frequent compared to classical chemotherapy based on available information on all adverse events related to the selected therapies.

## Methods

### FAERS adverse events data extraction

The FDA Adverse Event Reporting System obtains reports submitted to the FDA by the drug manufacturers, health care providers, and consumers [12]. All adverse events case reports pertaining to anti-PD1, anti-PD-L1, anti-CTLA4, and classical chemotherapy agents (listed in Additional File 1: Supplemental Table 2) and were extracted from Jan 2011-March 2020 in July 2020, using drug name keywords. Adverse events are entered using terms in the Medical Dictionary for Regulatory Activities (MedDRA) terminology (listed in Additional file 3) (http://www.meddramsso.com/index.asp). Case reports include information on causal medication, the reason for use, other medications currently in use, reaction, and case demographics.

### Data Processing & Statistical Analysis

We performed categorical classification for cases who received cardioprotective medications and/or oral steroids by text mining of medication keywords in the “Concomitant Product Names” column of the FAERS data using terms listed in Additional File 1: Supplemental Table 3. Cancer histology classification was performed according to the international classification of diseases (ICD9/10) for oncology and nomenclature of histologies (see Additional file 2). To overcome discrepancies of numerous terms or phrases used for the same cardiac adverse events, keywords for reactions were inspected and categorized to cardiac adverse event groups- myocarditis, pericarditis, myocardial infarction, coronary artery disease, arrhythmia, and heart failure (terms listed in Additional file 3). Text mining was performed using stringr R package (v1.4.0) [13]. The data pre-processing includes removal of outliers and missing variables for age, weight, and sex, cases of age < 18, as well as duplicates (Additional File 1: Supplemental Figure 1). The final study cohort included 90,740 cases. We performed a logistic regression analysis of matched cases (1:1) for sex, age, and weight. Case matching was performed using the MatchIt (v3.0.2) R package, and methods were set to “nearest” (nearest neighbor matching) [14]. Odds ratio and their corresponding 95% confidence intervals were calculated using the epiR (v1.0–15) and epiDisplay (v3.5.0.1) packages [15, 16]. Likelihood ratio test was used to calculate significance. Adjusted *p*-values with the threshold for statistical significance was set to *q* (adjusted p-value) <  0.05. The model was adjusted for age, weight, sex, oral steroid use, cardioprotective medication use, and chemotherapy use. Forest plots were created to display statistical summaries using the metafor R package (v2.4–0) [17]. All statistical analysis was performed in R (version 4.0.3).

## Results

### Elevated cardiovascular risk of immune checkpoint inhibitors

To evaluate the frequency of cardiac adverse events in patients treated with ICIs compared to classical chemotherapy, we performed a retrospective analysis using the FDA Adverse Event Reporting System. All adverse events case reports for anti-PD1, anti-PD-L1, anti-CTLA4 therapies, and classical chemotherapy agents were extracted in July 2020 (Additional File 1: Supplemental Table 2). Cohort demographics are displayed in Table 1. Following data processing and confounding factor adjustment (see Data Processing & Statistical Analysis Methods), the number of all adverse case reports was 90,740. For continuous variables, the mean and the interquartile range are displayed. Cardiovascular adverse events accounted for 9.1% of all adverse event (*n* = 8300). Anti-PD-(L)1, anti-CTLA4, and more than one ICI accounted for 20.4, 2.0, and 4.9% respectively; chemotherapy accounted for 72.6% of cases. We used logistic regression models to estimate the odds ratios (OR) and their 95% confidence intervals (CI) of matched cases, and found myocarditis and heart failure significantly correlated with ICIs and classical chemotherapy, respectively.

**Table 1:** FDA Adverse Event Reporting System analysis case demographics

Characteristics	n (%)
Total	90,740
Age	63 (53–70)
Sex	
Male	41,162 (45.4%)
Female	49,578 (54.6%)
Weight (kg)	73.5 (59.8–84.0)
Cardiac Adverse Events	8300 (9.1%)
Myocarditis	345 (0.4%)
Pericarditis	143 (0.2%)
Heart Failure	1706 (1.9%)
Myocardial Infarction	1594 (1.8%)
Arrhythmias	3858 (4.3%)
Coronary Artery Disease	654 (0.7%)
Anti-inflammatory medication use	18,797 (20.7%)
Cardioprotective medication use	23,372 (25.8%)
Treatment groups	
anti-PD-(L)1	18,536 (20.4%)
anti-CTLA4	1855 (2%)
combination immunotherapy	4442 (4.9%)
Chemotherapy	65,907 (72.6%)

PD-(L)1 = Programmed cell death protein -1 and Programmed death ligand -1 therapies; CTLA = cytotoxic T-lymphocyte-associated protein 4

**Fig. 1:**
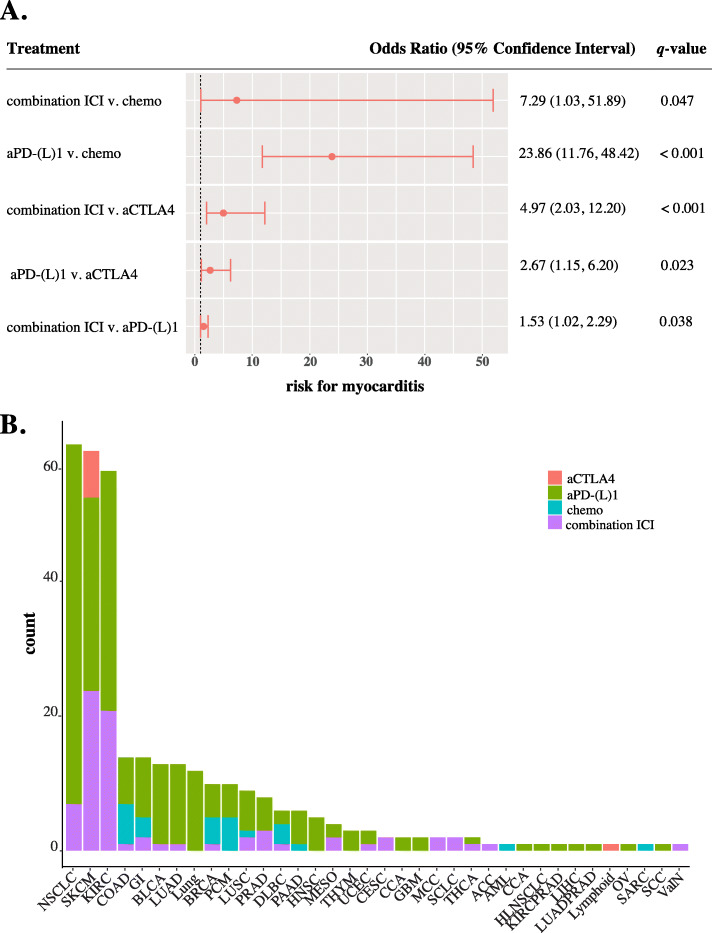
Immunotherapy is significantly associated with myocarditis. **a** Forest plot represents matched logistic regression model results for myocarditis of cancer treatment groups (anti-PD-(L)1, anti-CTLA4, combination (more than one ICI), and chemotherapy). Shown are significant adjusted *p*-value (*q* < .05) odds ratio and their 95% confidence interval. Red circles represent an odds ratio greater than one, favoring immunotherapy. Blue circles indicate an odds ratio less than one, favoring chemotherapy. **b** Incidence of myocarditis by cancer histology is shown. Each color represents which treatment group the case corresponded to. Red corresponds to anti-CTLA4, green to anti-PD-(L)1, blue to chemotherapy, and purple to combination immunotherapy. Anti-CTLA4 = anti-cytotoxic T-lymphocyte-associated protein 4; ICI = immune checkpoint inhibitor; anti-PD-(L)1 = anti-programmed death protein 1 and anti-programmed death-ligand 1

Using chemotherapy as a reference group, we found myocarditis was significantly higher in patients receiving combination immunotherapy, odds ratio (OR) =7.29 (95% confidence interval [CI] 1.03–51.89, *q* = 0.047), anti-program cell death protein 1 (PD-1), or anti-program death-ligand 1 (PD-L1), OR = 23.86 (95% confidence interval [CI] 11.76–48.42, *q* (adjusted *p*-value) < 0.001) We also found that combination immunotherapy presented a higher risk of myocarditis compared to anti-PD-(L)1, OR = 1.53 (95% CI 1.02–2.29, *q* = 0.038), and anti-CTLA4, OR = 4.97 (95% CI 2.03–12.20, *q* <  0.001), alone (Fig. 1a). This dataset included the most myocarditis cases (345) in respect to previous reports using the FAERS data. We did not observe a difference in the incidental risk of myocarditis between anti-PD-1 and anti-PD-L1 treatments, (Additional File 1: Supplemental Figure 2). We next evaluated the frequency of myocarditis among tumor histologies and found that non-small cell lung cancer (61 cases), melanoma (60 cases), and renal cell carcinoma (57 cases) had the most reports with all cases attributed to ICIs (Fig. 1b). Overall, we find that myocarditis is disproportionally more common following immunotherapy (Fig. 1, Additional File 1: Supplemental Table 1).

### Sex-specific cardiovascular risk of immune checkpoint inhibitors

**Fig. 2:**
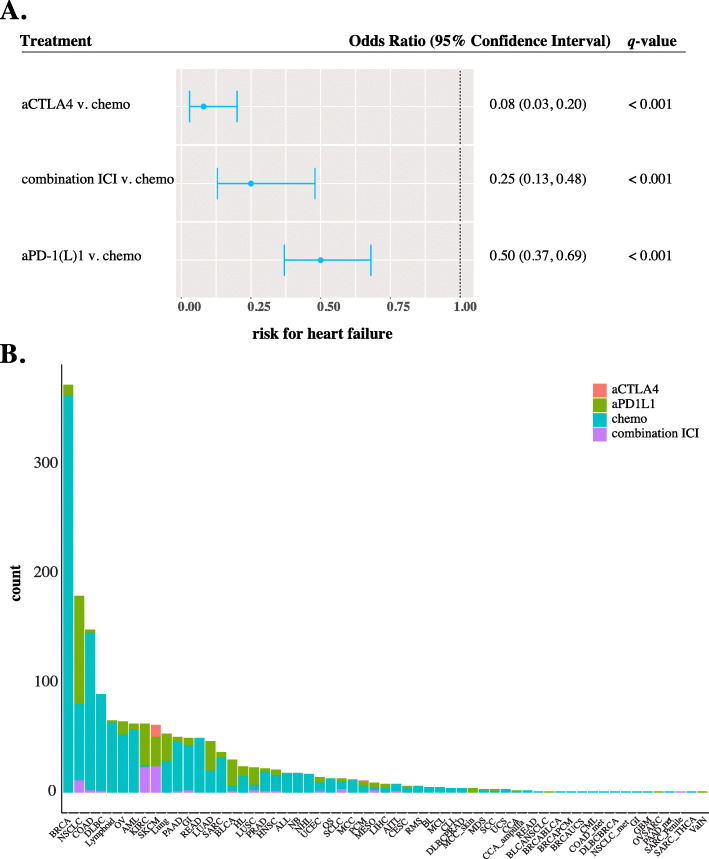
Heart failure adverse events in cancer therapy. **a** Forest plot represents matched logistic regression model results for heart failure in cancer treatment groups (anti-PD-(L)1, anti-CTLA4, combination (more than one ICI), and chemotherapy). Shown are significant adjusted p-value (*q* < .05) odds ratio and their 95% confidence interval. Red boxes represent an odds ratio greater than one, favoring immunotherapy. Blue boxes indicate an odds ratio less than one, favoring chemotherapy. **b** Incidence of heart failure by cancer histology is shown. Each color represents which treatment group the case corresponded to. Red corresponds to anti-CTLA4, green to anti-PD-(L)1, blue to chemotherapy, and purple to combination immunotherapy. Anti-CTLA4 = anti-cytotoxic T-lymphocyte-associated protein 4; ICI = immune checkpoint inhibitor; anti-PD-(L)1 = anti-programmed death protein 1 and anti-programmed death-ligand 1

**Fig. 3:**
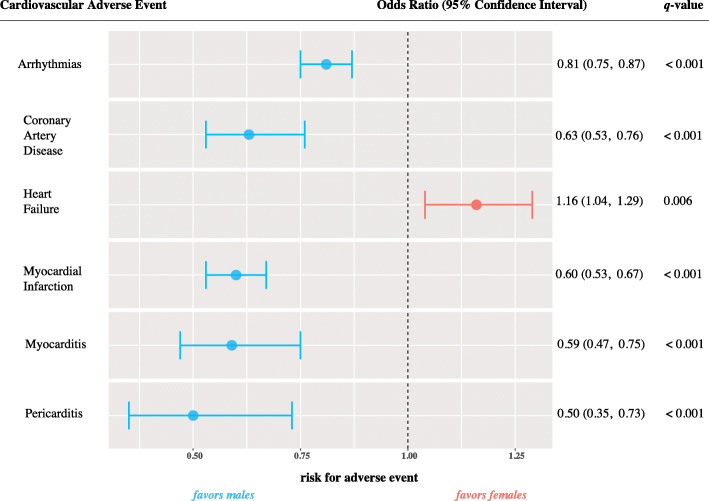
Sex-specificity related to cardiac adverse events. Forest plots represent multiple logistic regression model results for cardiac adverse events, using males as a reference. Shown are significant adjusted *p*-values (*q* < 0.05) odds ratio and their 95% confidence interval. Red circles indicate an odds ratio greater than one, favoring females; blue circles indicate an odds ratio less than one, favoring males

Using chemotherapy as a reference group, we found that the risk of heart failure was significantly lower in patients receiving anti-CTLA4, OR = 0.08 (95% CI 0.03–0.20, *q* <  0.001), anti-PD-(L)1, OR = 0.50 (95% CI 0.37–0.69, *q* <  0.001), and combination immunotherapy, OR = 0.25 (95% CI 0.13–0.48, *q* <  0.001) (Fig. 2a). Breast cancer represented the largest case reports for heart failure (373 cases), the majority of which were attributed to classical chemotherapy. We also report sex-specificity in females experiencing heart failure following chemotherapy, OR = 1.16 (95% CI 1.04–1.29, *q* = 0.006) (Fig. 2b, Fig. 3).

Recent interest has shed light on sex differences in numerous clinical phenotypes. We were interested to see whether sex differences extend to cardiac adverse events. Indeed, the following cardiac adverse events were more commonly reported in male individuals (odds ratio less than 1): myocarditis (OR = 0.59, 95% CI 0.47–0.75, *q* <  0.001), pericarditis (OR = 0.50, 95% CI 0.35–0.73, *q* <  0.001), myocardial infarction (OR = 0.60, 95% CI 0.53–0.67, q <  0.001), coronary artery disease (OR = 0.63, 95% CI 0.53–0.76, *q* <  0.001), and arrhythmias (OR = 0.81, 95% CI 0.75–0.87, *q* <  0.001) (Fig. 3).

We also performed a logistic regression analysis of unmatched cases. We observed that chemotherapy had a higher risk for arrhythmias compared to combination immunotherapy, OR = 0.71 (95% CI 0.61–0.84, *q* <  0.001), and anti-PD-(L)1, OR = 0.67 (95% CI 0.61–0.73, *q* <  0.001) therapy (Table 2). However, when testing the effect of individual confounders on model performance, we do not see that the use of cardioprotective medications correlated with arrhythmia adverse events (OR = 1.0, 95% CI 0.93–1.1, *q* = 0.955). We also show chemotherapy has a higher risk for myocardial infarction compared to PD-(L)1 monotherapy (OR = 0.44, 95% CI 0.34–0.58, *q* <  0.001), CTLA-4 monotherapy (OR = 0.56, 95% CI 0.36–0.85, *q* = 0.007), and combination immunotherapy (OR = 0.42, 95% CI 0.29–0.6, *q* <  0.001). Overall, our analysis reveals sex differences in the risk of cardiac adverse events in cancer patients.

**Table 2:** Complete analysis of all cardiac AE risk from immunotherapy versus chemotherapy

Adverse Event	Therapy	***OR (95% CI)***	***q-value***
Pericarditis	Combination v chemo	0.61 (0.23, 1.67)	0.339
	Combination v aCTLA4	1.79 (0.39, 8.3)	0.457
	Combination v aPD1L1	0.48 (0.24, 0.97)	0.04
	aPD1L1 v chemo	1.28 (0.6, 2.72)	0.523
	aCTLA4 v chemo	0.34 (0.07, 1.66)	0.184
	aCTLA4 v aPD1L1	0.27 (0.07, 1.1)	0.068
Myocardial Infarction	Combination v chemo	0.42 (0.29, 0.6)	< 0.001
	Combination v aPD1L1	0.94 (0.72, 1.24)	0.671
	Combination v aCTLA4	0.75 (0.5, 1.14)	0.178
	aPD1L1 v chemo	0.44 (0.34, 0.58)	< 0.001
	aCTLA4 v chemo	0.56 (0.36, 0.85)	0.007
	aCTLA4 v aPD1L1	1.25 (0.87, 1.79)	0.219
Heart Failure	Combination v chemo	0.38 (0.27, 0.54)	< 0.001
	Combination v aPD1L1	0.84 (0.65, 1.08)	0.171
	Combination v aCTLA4	2.21 (1.22, 3.9)	< 0.001
	aPD1L1 v chemo	0.46 (0.36, 0.59)	< 0.001
	aCTLA4 v chemo	0.17 (0.1, 0.32)	< 0.001
	aCTLA4 v aPD1L1	0.38 (0.22, 0.66)	< 0.001
Coronary Artery Disease	Combination v chemo	0.64 (0.37, 1.12)	0.12
	Combination v aCTLA4	1.47 (0.59, 3.63)	0.405
	Combination v aPD1L1	0.96 (0.6, 1.52)	0.848
	aPD1L1 v chemo	0.67 (0.47, 0.96)	0.028
	aCTLA4 v chemo	0.44 (0.18, 1.06)	0.066
	aCTLA4 v aPD1L1	0.65 (0.28, 1.49)	0.309
Arrhythmias	Combination v CTLA4	0.92 (0.7, 1.23)	0.584
	Combination v chemo	0.71 (0.61, 0.84)	< 0.001
	Combination v aPD1L1	1.06 (0.89, 1.26)	0.531
	aPD1L1 v chemo	0.67 (0.61, 0.73)	< 0.001
	aCTLA4 v chemo	0.77 (0.61, 0.98)	0.035
	aCTLA4 v aPD1L1	1.15 (0.89, 1.47)	0.289
Myocarditis	Combination v aPD1L1	1.41 (1.08, 1.85)	0.012
	Combination v chemo	30.76 (18.42, 51.37)	< 0.001
	Combination v aCTLA4	3.93 (1.89, 8.16)	< 0.001
	aPD1L1 v chemo	21.81 (13.89, 34.24)	< 0.001
	aCTLA4 v chemo	7.83 (3.41, 18)	< 0.001
	aCTLA4 v aPD1L1	0.36 (0.18, 0.73)	0.005

Shown is the logistic regression model data of the entire dataset, cases are not matched; Combination indicates more than one immunotherapy was administered. Adjusted p-values (*q*-value) is displayed

aPD1L1 = anti- Programmed cell death protein - 1 and Programmed death ligand -1; aCTLA4 = cytotoxic T-lymphocyte- associated proteni 4

## Discussion

Our study provides comprehensive risk assessments of cardiac adverse events reports in cancer patients treated with immunotherapy and/or chemotherapy and includes 8300 cardiac adverse events reports. Although rare, cardiac adverse effects are shown to lead to serious consequences. Using the World Health Organization pharmacovigilance database, Wang et al. identified that cardiac and neurological events represent one-third of immune checkpoint inhibitor-related fatalities. The proportion of immune-related fatalities were more frequent (21%) in cases receiving more than one immunotherapy drug [11]. Among cardiac adverse events, others have found that ICI-associated myocarditis has a mortality risk of 50% [11, 18]. Here, we validate that myocarditis remains disproportionally more common following ICI therapy compared to chemotherapy. This evidence reflects prior estimates of myocarditis using external databases (< 1%) [11, 19, 20]. We also show that within this dataset myocarditis is more frequent in males. These findings follow previous reports of myocarditis occurring more frequently in men [11]. Furthermore, our analysis shows myocarditis reports were largely from patients with non-small cell lung cancer, melanoma, and renal cell carcinoma. This is likely a reflection of the cancer histologies receiving earlier FDA approval for immunotherapy. Despite higher surveillance of myocarditis leading to more reports in the last years, we find that myocarditis is disproportionally more common following immunotherapy (Fig. 1, Additional File 1: Supplemental Table 1).

The cause of interindividual variability in immunotherapy-mediated cardiotoxicity remains unclear. Clinical case reports have shown fatal cases of fulminant myocarditis following a single dose of anti-PD1 or anti-CTLA4 treatment. Martinez-Calle et al. reported patients tested positive for cardiac troponin auto-antibodies and had elevated troponin T levels suggesting a pre-existing T memory response that was abrogated by PD-1 blockade [21]. T cell receptor recognition in the heart thus results in a cytotoxic effect on cardiac tissue. Johnson et al. reported the presence of infiltrating lymphocytes and macrophages in the cardiac muscle. Lymphocyte receptor sequencing showed a significant overlap of TCR sequences among cardiac, skeletal, and tumor infiltrates, suggesting that the antigens in the myocardium and skeletal muscle were recognized by infiltrating lymphocytes [20]. However, we still do not fully understand which risk factors that may predispose a patient to lethal cardiac adverse events. Mechanistic studies to identify causality are imperative and underway.

Some opponents argue that the need for adding additional medical assessments for rare adverse events is an unnecessary burden for both patients and health care providers. Ederhy et al. highlighted clinical trials from 2010 to 2016 and reported only 15 cases of cardiac adverse events. A caveat to consider, however, is the lack of inclusion of patients with a medical history of cardiovascular disease and the lack of monitoring for cardiac related toxicity in ICI clinical trials [22]. Needless to say, pharmacovigilance reports have been critical in raising awareness and increasing surveillance. As a result, guidelines on clinical assessment, medical testing, intervention, and surveillance have been drafted to address this medical niche [23, 24]. Since 2019, FAERS database reports of all cardiac adverse events have increased and now account for 50–60% of all cardiac adverse events in immunotherapy-related reports (Additional File 1: Supplemental Table 1). For this reason, we provide an updated analysis, to include a comparison with chemotherapy-related adverse events reports.

In this study, we also report a greater risk for heart failure in chemotherapy-treated patients. We observed that breast cancer represents the largest cancer histology among cases reported for heart failure. This finding likely reflects the well-established correlation of anthracycline-induced heart failure in breast cancer. Anthracycline-induced cardiotoxicity can occur as acute toxicity that results in arrhythmias or depressed left ventricular ejection fraction (LVEF), or chronic toxicity which results from excessive exposure or concomitant risk factors. The risk for developing heart failure increases with a high cumulative dose of doxorubicin [25]. However, only about half of patients show adverse effects at higher doses, indicating that heritable features might play a role in drug exposure, efficacy, and response [26]. A caveat to consider, reports of heart failure in breast cancer occur several years following drug exposure. With limited longitudinal data of immunotherapy since its initial approval in 2011, it may be too early to test whether heart failure is detected following ICI exposure [27].

We also report that immune-mediated cardiac adverse events are disproportionately more frequent in males. Several factors can lend to this observation. The occurrence of immune-related AEs is an indicator of ICI activity. Males are reported to have a better treatment effect of ICIs as reported in a meta-analysis from 20 randomized controlled trials [28]. More specifically, results from the KEYNOTE-024 Phase 3 trial testing the efficacy of pembrolizumab (anti-PD-1) in non-small cell lung cancer found that males exhibited a lower hazard for disease progression (HR = 0.39, 95% CI 0.26–0.58), compared to females (HR = 0.75, 95% CI 0.46–1.21) [29]. Recent reports have investigated biological determinants of sex-specific responses to immunotherapy. Castro et al. have reported that sex differences relate to females exhibiting a strong immune selection early in tumorigenesis, which in turn leads to fewer driver mutations visible to the immune system at the time of ICI treatment [30]. This postulation corresponds well with recent reports from population GTEx data showing sex-biased expression of immune-related pathways (antigen-presentation and T-cell proliferation) in females [31]. These compelling findings underscore the need to consider efficacy and risk assessment differently in males and females.

## Outlook

Comprehensive identification of patients’ specific predispositions, to individualize immunotherapy strategies and therefore yield the greatest clinical benefits at the lowest impact of heart and cardiovascular systems, is the ultimate goal of precision cardio-oncology and immunotherapy. Data mining of the large population registries, like the FAERS database, are important tools that allow for understanding clinical features correlated to drug adverse outcomes. However, such approaches will not be sufficient to identify causality. More comprehensive databases can further enrich our understanding of drug outcomes using pharmacogenomic approaches. The Clinical Pharmacogenetics Implementation Consortium, PharmGKB, and the RadioGenomics Consortium are examples of consortia that are focusing on evaluating genetic determinants of drug responses and adverse effects. Additionally, they help set guidelines for translating genotyping tests in cancer patients receiving treatment [32–34]. Individual national and hospital registries are also reporting major cardiovascular events using electronic health care data following cancer treatment [8, 35–38]. These studies are important for validating AE incidence determined in pharmacovigilance studies and help identify additional important clinical determinants, such as the time of AE following drug exposure.

The next wave of health data availability will require advanced computational approaches for interpretation and medical applications. Artificial intelligence (AI) approaches to interpreting large-scale electronic health care record data, with machine learning algorithms will be critical to identify important features associated with cardiovascular adverse events [39]. To date, pioneering applications of artificial intelligence in the field of cardiology have focused on the interpretation of electrophysiology and cardiovascular imaging data [40–42]. Recently, Zhou et al. applied a durable machine learning model to assess six distinct cancer therapy-related cardiac events in 4309 cancer patients. The results identified 23 clinically relevant variables for at least one cardiac AE [39]. These research studies will be imperative to enable preventive measures of cardiac events in cancer patients. However, the precision of AI models’ prediction and interpretation is limited by the quality of data, scale of the cohort, and availability and efficiency of computational resources [40].

## Study limitations

We acknowledge several possible limitations in the current study, including heterogeneity and bias in self-reporting. We are not able to report risk estimates, given that our statistical analysis did not include cancer patients who did not experience adverse events following immunotherapy and, and thus we also cannot explain causality. We are not able to account for the probability of duplicate cases reported by health care professionals or the drug manufacturer and consumers. Although our statistical models adjusted for confounding factors, unmeasured confounding factors may exist as well. For example, the association of a drug with an adverse event might be explained by those of drugs that are co-administered. We also do not have information on cancer stage, or time of report following drug exposure, which could affect the associations. The database also lacks an indication of the medical history of cardiovascular disease prior to drug exposure. To overcome this limitation, we adjusted our model for the use of cardioprotective medications. Randomized controlled prospective studies will be imperative to validate findings, and ought to become clearer whether events are either a direct insult from cancer treatment or the exacerbation of underlying disease comorbidities (i.e., diabetes, hypertension, and existing cardiovascular conditions).

## Conclusion

Here we report an elevated incidence of myocarditis in patients receiving immunotherapy compared to chemotherapy. Furthermore, we find that males are disproportionately at higher risk of immune-related cardiac adverse events. Additional clinical features can help expand the risk stratification of AE following ICI treatment. Understanding the clinical risk factors that predispose ICI-treated cancer patients to cardiotoxicity will be crucial in Cardio-Oncology management.

## Supplementary Information

**Additional file 1: Supplementary Figures.**

**Additional file 2. Tumor Histology acronyms.**

**Additional file 3. Adverse Events keywords.**

## Data Availability

The datasets used and/or analysed during the current study are available from the FDA Adverse Event Reporting System (FAERS) Public Dashboard [12].
