# Supplementary material for: A retrospective analysis of cardiovascular adverse events associated with immune checkpoint inhibitors
Source: Cardiooncology. 2021 May 28;7:19. doi: 10.1186/s40959-021-00106-x (PMC8161966; doi:10.1186/s40959-021-00106-x)
Supplement: Supplementary file 1 — Additional file 1: Supplementary Figures. [file 40959_2021_106_MOESM1_ESM.pdf]

Supporting Information

Title: A Retrospective Analysis of Cardiovascular Adverse Events Associated with Immune Checkpoint Inhibitors

Jessica Castrillon Lal[1,2], Sherry-Ann Brown[3], Patrick Collier[2,4], Feixiong Cheng[1,2,5,*]

[1]Genomic Medicine Institute, Lerner Research Institute, Cleveland Clinic, Cleveland, OH 44195, USA

[2]Department of Molecular Medicine, Cleveland Clinic Lerner College of Medicine, Case Western Reserve University, Cleveland, OH 44195, USA

[3]Cardio-Oncology Program, Division of Cardiovascular Medicine, Medical College of Wisconsin, Milwaukee, WI 53226, USA

[4]Robert and Suzanne Tomsich Department of Cardiovascular Medicine, Sydell and Arnold Miller Family Heart and Vascular Institute, Cleveland Clinic, Cleveland, OH 44195, USA

[5]Case Comprehensive Cancer Center, Case Western Reserve University School of Medicine, Cleveland, Oh 44106, USA

*Correspondence to:

Feixiong Cheng, PhD

Lerner Research Institute, Cleveland Clinic

Tel: +1-216-444-7654; Fax: +1-216-636-0009

Email: chengf@ccf.org

The Supporting Information includes 3 Supplemental Tables (pdf), 5 Supplemental Figures (pdf), and two additional files (excel file).

Supplemental Table 1. Incidence of all cardiac adverse event reports in the FAERS database.

Immunotherapy	< 2017	2018	2019	2020	cardiac AE in 2019-2020/ all cardiac AE (%)
Avelumab	11	22	33	20	61.6
Atezolizumab	74	118	223	171	67.2
Durvalumab	21	58	79	40	60.1
Ipilimumab	455	187	260	236	43.6
Pembrolizumab	287	310	363	243	50.4
Nivolumab	930	614	778	533	45.9
Cemiplimab	0	2	12	6	90.0

Supplemental Table 2. Cancer therapy keywords used for extracting adverse events reports from FAERS database.

Platinum	***Topoisomerase Inhibitor***	***Anthracyclines***	***Cytotoxic Drugs***	***Immunotherapy***
Carboplatin	Camptothecin	Doxorubicin	Bleomycin	Atezolizumab
Cisplatin	Irinotecan	Daunorubicin	Clofarabine	Avelumab
Oxaliplatin	Topotecan		Rigosertib	Durvalumab
				Ipilimumab
Taxol	***DNA Intercalator***	***Antimetabolite***		Cemiplimab
Docetaxel	Etoposide	Gemcitabine		Nivolumab
Paclitaxel	Teniposide	Methotrexate		Pembrolizumab
Vincristine		Pemetrexed		
		Fluorouracil		

Supplemental Table 3. Cardioprotective and anti-inflammatory medications used for model correction.

Beta-blocker	***Calcium Channel Blocker***	***Alpha-adrenergic Blocker***	***Angiotensin II Agonist***	***Diuretic***	***Anticoagulant***
Nebivolol	Amlodipine	Doxazosin	Candesartan	Spironolactone	Aspirin
Timolol	Clevidipine	Phenoxybenzamine	Irbesartan	Torsemide	Clopiogrel
Carvedilol	Diltiazem	Prazosin	Olmesartan	Chlorothiazide	Dipyridamole
Nadolol	Felodipine	Terazosin	Losartan	Methyclothiazide	Prasugrel
Propanolol	Isradipine	Clonidine	Valsartan	Hydrochlorothiazide	Ticagrelor
Betaxolol	Nicardipine	Guanfacine	Azilsartan	Furosemide	Warfarin
Penbutolol	Nimodipine		Telmisartan	Indapamide	
Metoprolol	Nisoldipine		Eeprosartan	Hydroflumethiazide	
Acebutolol	Verapamil			Chlorothalidone	
Atenelel				Metolazone	
Labetalol					
Pindolol					
Bisoprolol					
Oral steroid		***Vasodilator***	***Renin Inhibitor***	***Glycoside***	
Dexamethasone		Hydralazine	Aliskiren	Digoxin	
Hydrocortisone		Minoxidil		Lanoxin	
Prednisone					
Deltasone					
Meprednisone					
Methylprednisone					
Prednisolone					

Supplemental Figures

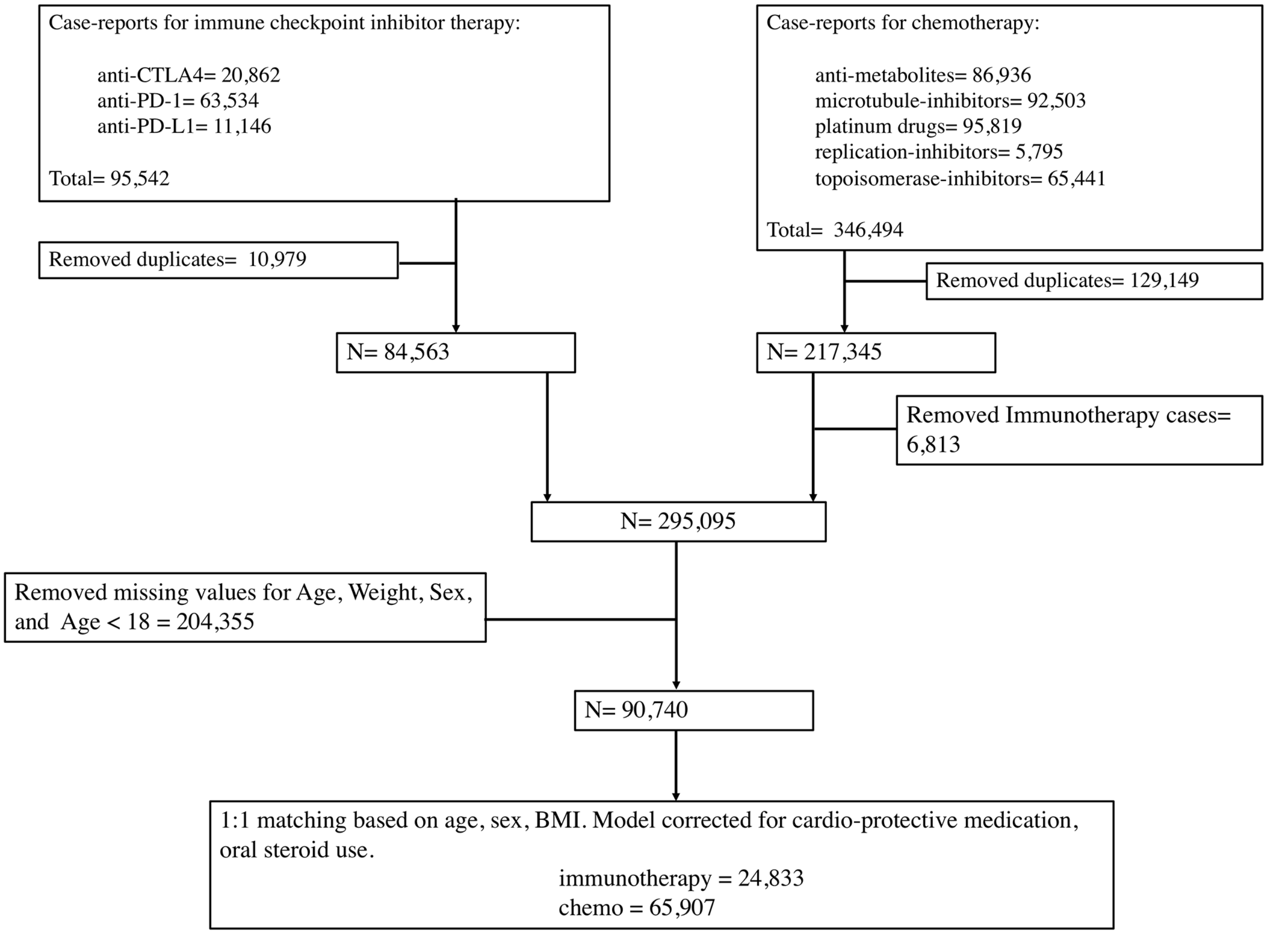

Supplemental Figure 1. Strategy of data extraction and quality assessment. Data extraction is described in the methods. Shortly, FDA Adverse events reports were queried by drug name. Duplicate reports were removed. All chemotherapy reports that included concomitant drugs with immunotherapy were removed. Cases with missing values for age, weight, sex were removed. As well as cases younger than 18. Immunotherapy and chemotherapy cases were matched by age, weight, and sex. Model was also corrected for cardio-protective medication and oral-steroid use.

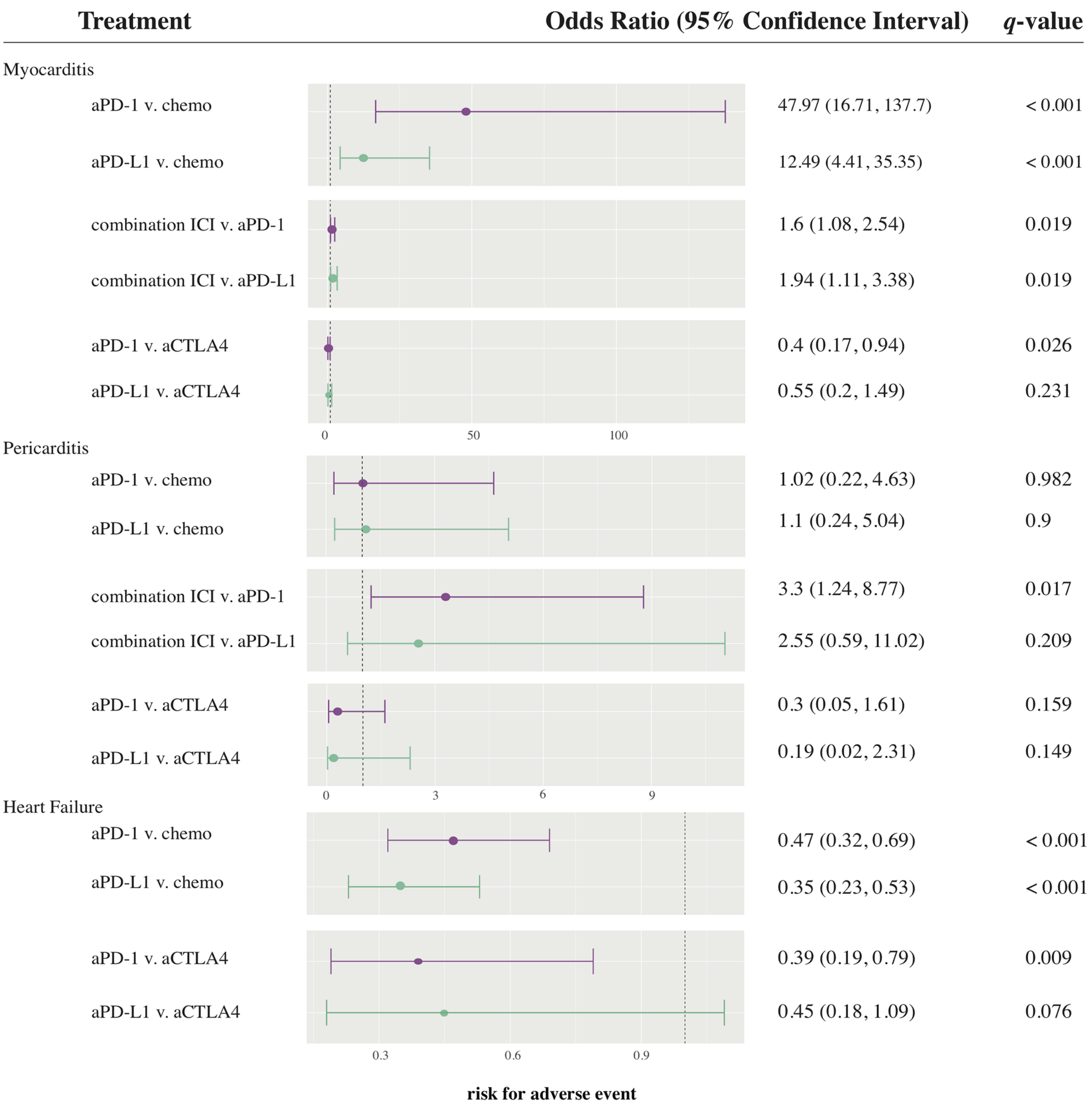

Supplemental Figure 2. Risk differences of myocarditis from anti-PD-1 and anti-PD-L1 immunotherapies. Forest plot represents matched logistic regression model results for myocarditis adverse events comparing differences in outcomes of anti-PD-1 and anti-PD-L1 therapies with anti-CTLA4, combination (more than one ICI), and chemotherapy). Shown are the odds ratio (OR), their 95% confidence interval, and adjusted p-values (*q*). Red boxes indicate an odds ratio greater than one favoring the first treatment listed, blue boxes indicate an odds ratio less than one favoring the second treatment listed. Combination treatment indicates more than one immunotherapy administered. Of note, no significant differences were detected between anti-PD-1 and anti-PD-L1 therapy.

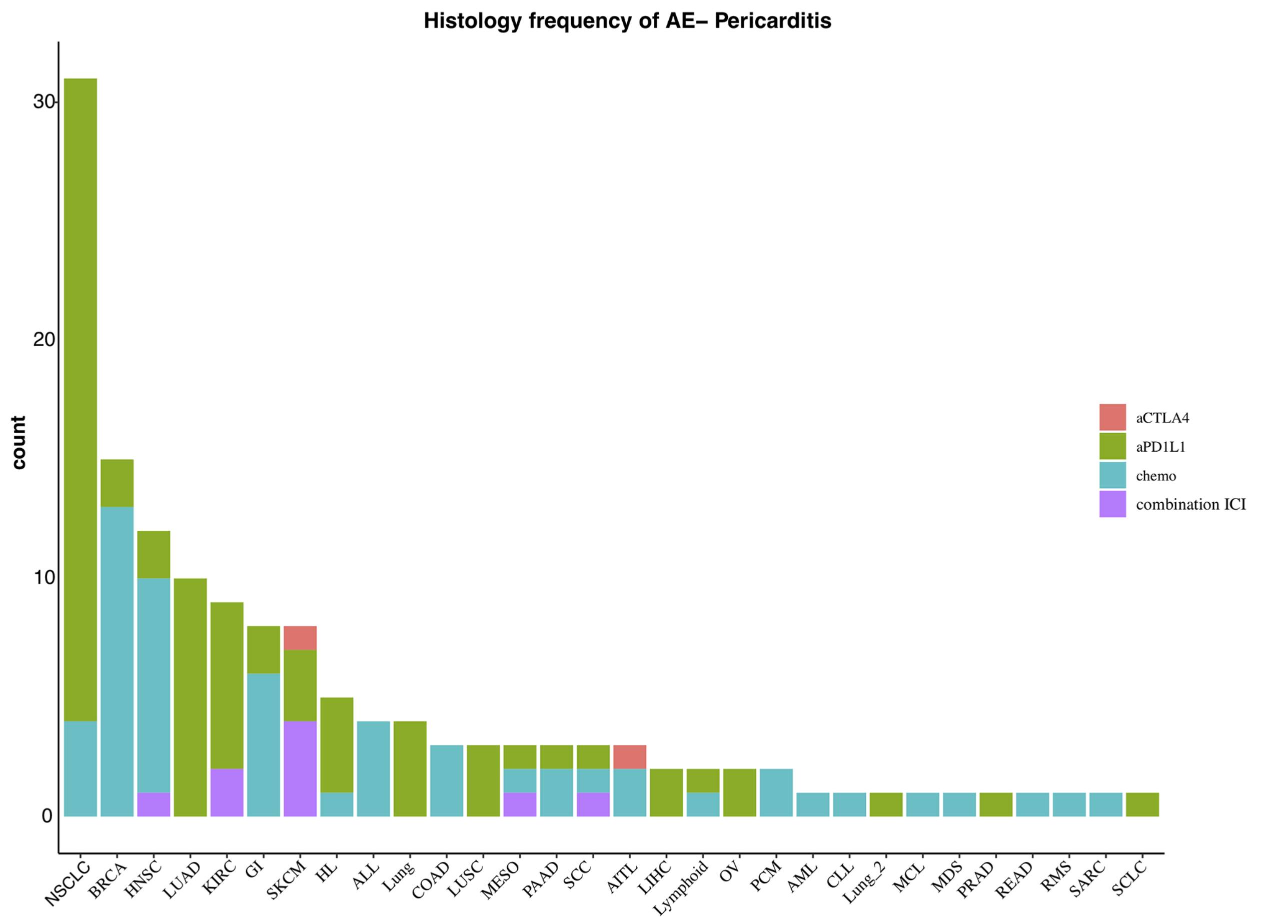

Supplemental Figure 3. Other cardiac AE stratified by cancer histology and treatment group. Incidence of pericarditis by cancer histology is shown. Each color represents which treatment group the case corresponded to. Red corresponds to anti-CTLA4, green to anti-PD-(L)1, blue to chemotherapy, and purple to combination immunotherapy.

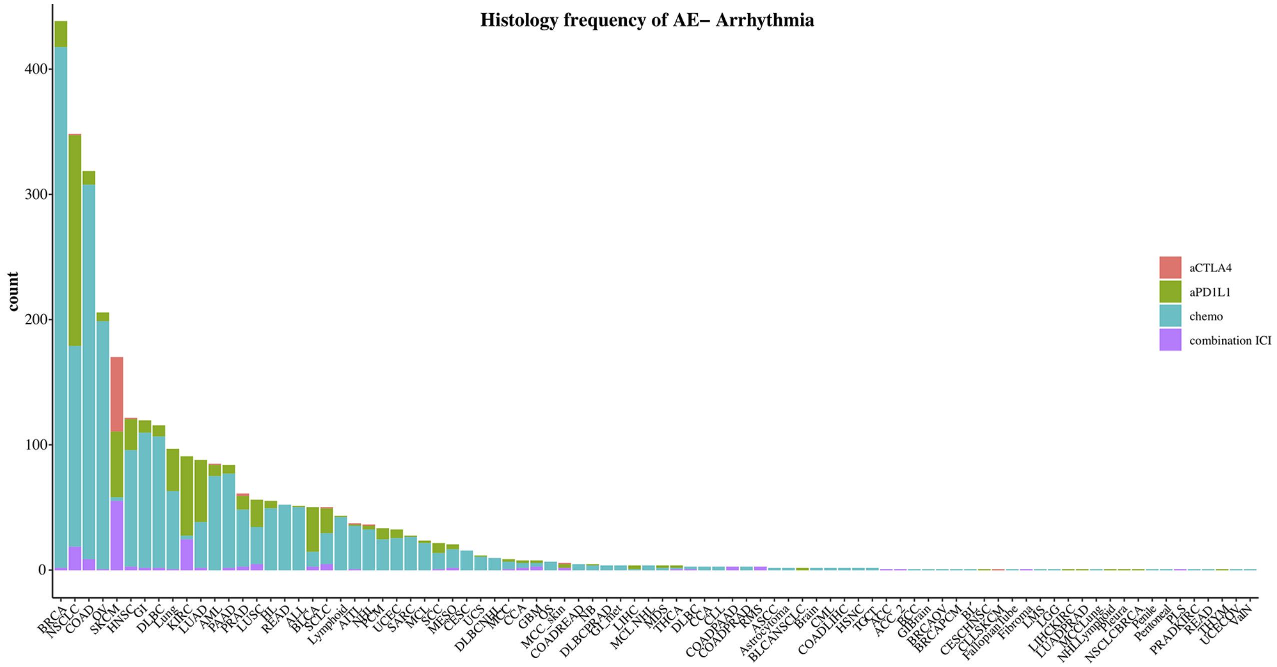

Supplemental Figure 4. Other cardiac AE stratified by cancer histology and treatment group. Incidence of arrhythmias by cancer histology is shown. Each color represents which treatment group the case corresponded to. Red corresponds to anti-CTLA4, green to anti-PD-(L)1, blue to chemotherapy, and purple to combination immunotherapy.

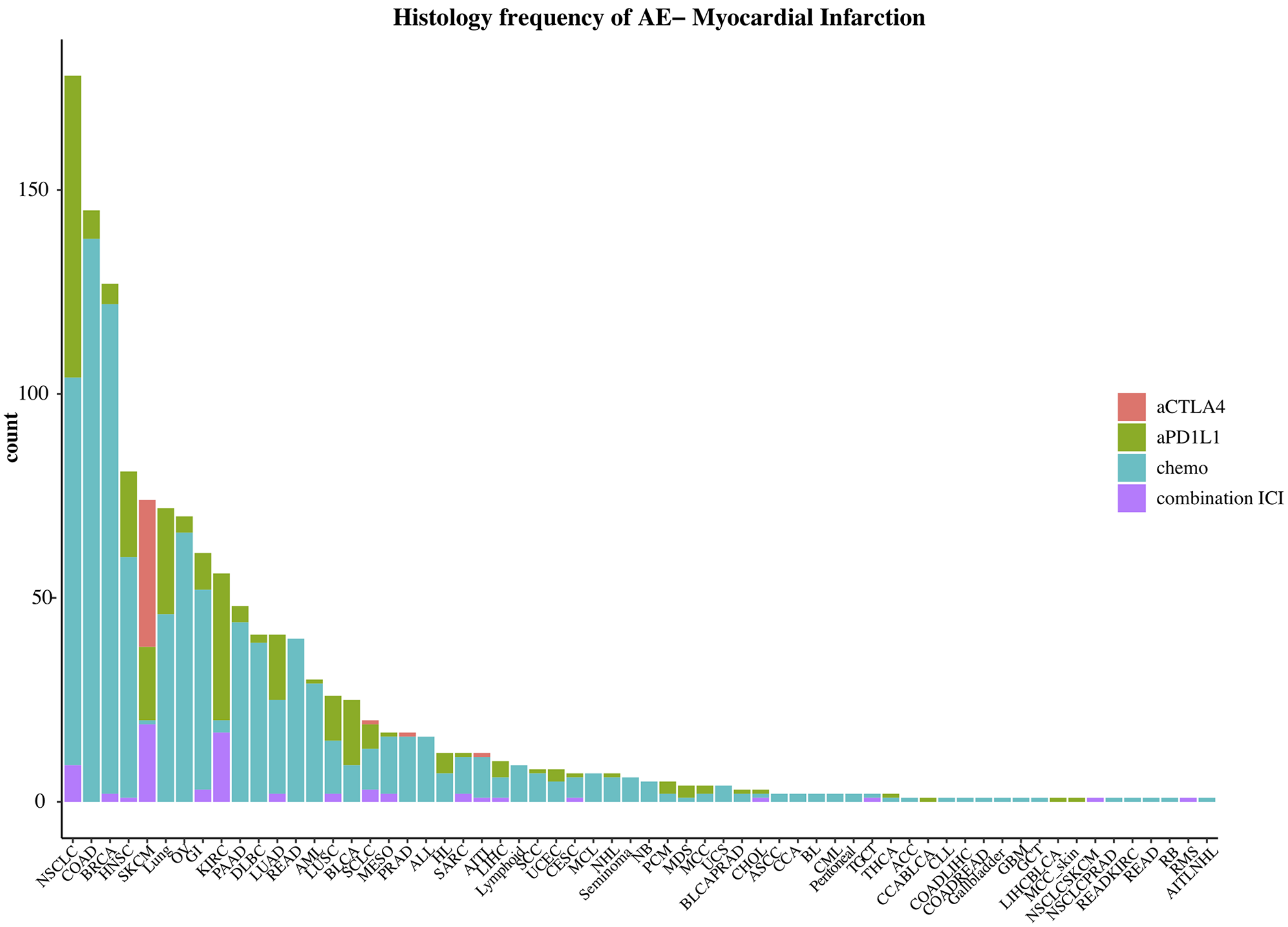

Supplemental Figure 5. Other cardiac AE stratified by cancer histology and treatment group. Incidence of myocardial infarction by cancer histology is shown. Each color represents which treatment group the case corresponded to. Red corresponds to anti-CTLA4, green to anti-PD-(L)1, blue to chemotherapy, and purple to combination immunotherapy.
